# ICTV Virus Taxonomy Profile: Hytrosaviridae

**DOI:** 10.1099/jgv.0.001300

**Published:** 2019-08-07

**Authors:** Henry M. Kariithi, Just M. Vlak, Johannes A. Jehle, Max Bergoin, Drion G. Boucias, Adly M. M. Abd-Alla, ICTV Report Consortium

**Affiliations:** 1 Biotechnology Research Institute, Kenya Agricultural and Livestock Research Organization, Nairobi 00200, Kenya; 2 Laboratory of Virology, Wageningen University and Research, Wageningen 6708 PB, The Netherlands; 3 Institute for Biological Control, Federal Research Centre for Cultivated Plants, Julius Kühn-Institut, Darmstadt 64287, Germany; 4 Laboratoire de Pathologie Comparée, Faculté des Sciences, Université de Montpellier, Montpellier 34095, France; 5 Entomology and Nematology Department, University of Florida, Gainesville FL 32611, USA; 6 Insect Pest Control Laboratory, Joint FAO/IAEA Division of Nuclear Techniques in Food and Agriculture, Vienna A-1400, Austria

**Keywords:** *Hytrosaviridae*, ICTV Report, taxonomy

## Abstract

*Hytrosaviridae* is a family of large, rod-shaped, enveloped entomopathogenic viruses with dsDNA genomes of 120–190 kbp. Hytrosaviruses (also known as salivary gland hypertrophy viruses) primarily replicate in the salivary glands of adult dipteran flies. Hytrosaviruses infecting the haematophagous tsetse fly and the filth-feeding housefly are assigned to two genera, *Glossinavirus* and *Muscavirus,* respectively. Whereas muscavirus infections are only overt, glossinavirus infections can be either covert or overt. Overt infections are characterized by diagnostic salivary gland hypertrophy and cause either partial or complete infertility. This is a summary of the International Committee on Taxonomy of Viruses (ICTV) Report on the family *Hytrosaviridae*, which is available at ictv.global/report/hytrosaviridae.

## VIRION

**Fig. 1.**
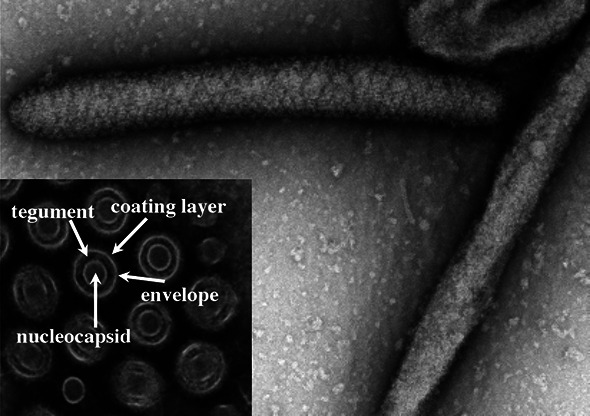
Transmission electron micrographs showing the structural features of Glossina pallidipes salivary gland hypertrophy virus – Uganda strain; inset – cross-section. Adapted from [5].

Hytrosaviruses have non-occluded, enveloped, rod-shaped virions measuring 50–80×500–1000 nm, which contain a thin, dense, central nucleocapsid encasing the DNA–protein core (Table 1, Fig. 1) [1,4]. The outer surface of glossinavirus virions is studded with left-handed helical polymeric spikes (13 nm long; 15 nm periodicity) composed of virus and host-derived protein dimers (23 spikes×24 helical turns=1104 envelope dimers). A 10 nm-thick amorphous proteinaceous tegument surrounds the 40 nm diameter helical nucleocapsid core underlying the virus envelope [3,4]. Muscavirus virions contain regularly braided, bead-like surface projections [2,5].

**Table 1.** Characteristics of members of the family *Hytrosaviridae*

Typical member:	Glossina pallidipes salivary gland hypertrophy virus (EF568108), species *Glossina hytrovirus*, genus *Glossinavirus*
Virion	Typically, enveloped particles of 50–80×500–1000 nm
Genome	Circular, dsDNA, 120–190 kbp, encoding 108–174 proteins
Replication	DNA synthesis and transcription within nuclear replication complexes; temporal expression of genes
Translation	Presumably via cap-dependent, polyadenylated monocistronic mRNAs
Host range	Dipterans: tsetse fly (*Glossinavirus*); housefly and stable fly (*Muscavirus*)
Taxonomy	Two genera (*Glossinavirus* and *Muscavirus*)

## Genome

**Fig. 2.**
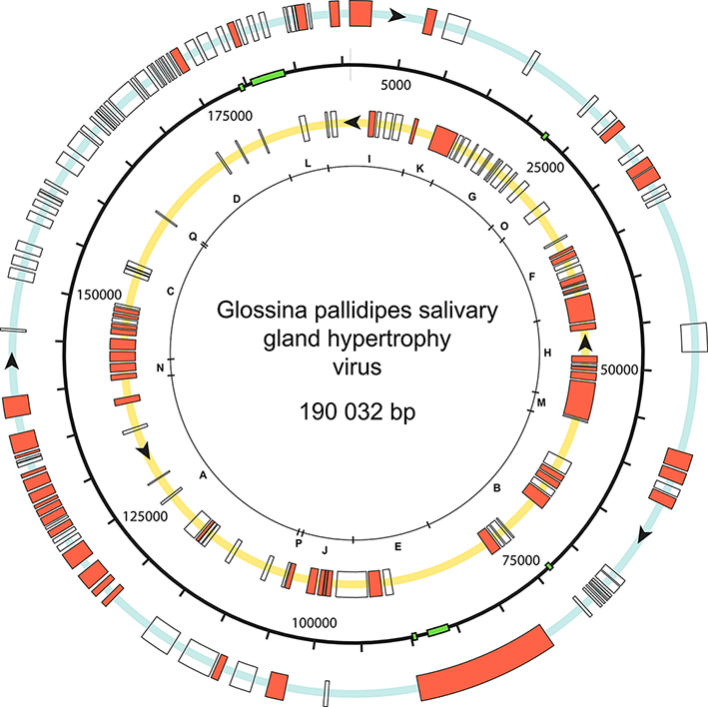
Genome of Glossina pallidipes salivary gland hypertrophy virus (Ethiopia strain). Putative open reading frames are indicated by boxes on the blue (clockwise transcription) or yellow (anti-clockwise transcription) circles, with those encoding virion proteins indicated by salmon colour. The letters on the innermost ring represent BglII restriction fragments. Green boxes indicate the position of direct repeat sequences. Information derived from [6].

Hytrosaviruses contain large, supercoiled, circular dsDNA genomes of 120–190 kbp with 108–174 putative, non-overlapping genes that are evenly distributed over both DNA strands in unidirectional clusters (Fig. 2) [6]. The transcription elements of the muscavirus genome are largely unknown, but the majority of the ORFs are enriched with TAAG motifs, poly(A) signals and TATA box elements [5]. At least 61 glossinavirus ORFs and 29 muscavirus ORFs are known to encode virion proteins.

## Replication

Hytrosaviruses primarily replicate in virogenic stroma in the nuclei of salivary gland cells, and in non-salivary gland tissues (e.g. tracheal cells, milk glands, corpora allata/cardiaca). Following internalization, capsids are released into the cytoplasm and traffic to the cell nucleus, where gene transcription, DNA replication and nucleocapsid assembly in the virogenic stroma occurs [4,7]. Virus replication is thought to involve the expression of immediate early (transcription factor), early (DNA replication) and late (structural protein) genes [4,5]. Nucleocapsids exit the nucleus via the nuclear pore complex and acquire envelopes in the cytoplasm; mature particles egress by budding through (muscavirus virions) or lysis (glossinavirus virions) of the luminal membranes of infected cells [4,5]. Replication in non-salivary gland tissues causes partial sterility (in tsetse fly) and complete shutdown of vitellogenesis (in housefly) [8]. Hytrosaviruses presumably produce capped, polyadenylated, monocistronic mRNAs, and possibly use cap-dependent translation [4,6].

## Taxonomy

Two species in two genera have been described: *Glossina hytrovirus* in *Glossinavirus* and *Musca hytrovirus* in *Muscavirus* [1]. A future Taxonomic Proposal will seek to align the spelling of species names with that of the family. A related, unclassified virus infects the phytophagous syrphid fly, *Merodon equestris* [9]. Phylogenetic analysis of virus DNA polymerases indicates clearly that members of the *Hytrosaviridae* are distant from other viruses with large DNA genomes.

## Resources

Full ICTV Report on the family *Hytrosaviridae*: ictv.global/report/hytrosaviridae.
